# Reproducibility and relative validity of a semi-quantitative food frequency questionnaire for the Chinese lactating mothers

**DOI:** 10.1186/s12937-021-00678-5

**Published:** 2021-03-03

**Authors:** Ye Ding, Fang Li, Ping Hu, Mei Ye, Fangping Xu, Wei Jiang, Yue Yang, Youjuan Fu, Yunhua Zhu, Xiaolong Lu, Ying Liu, Zhencheng Xie, Zhixu Wang

**Affiliations:** 1 Department of Maternal, Child and Adolescent Health, School of Public Health, Nanjing Medical University, Nanjing, Jiangsu 211166 People’s Republic of China; 2 Nanjing Jiangning District Maternal and Child Health and Family Planning Service Center, Nanjing, Jiangsu 211100 People’s Republic of China

**Keywords:** Food frequency questionnaire, Reproducibility, Relative validity, Lactating mothers, Diet, China

## Abstract

**Background:**

The dietary nutritional status of the lactating mothers is related to maternal health and has a significant impact on the growth and development of infants through the secretion of breast milk. The food frequency questionnaire (FFQ) is the most cost-effective dietary assessment method that can help obtain information on the usual dietary pattern of participants. Until now, the FFQs have been used for different populations in China, but there are few FFQs available for the lactating mothers. We aimed to develop a semi-quantitative, 156-item FFQ for the Chinese lactating mothers, and evaluate its reproducibility and relative validity.

**Methods:**

A total of 112 lactating mothers completed two FFQs and one 3-d dietary record (3DR). The first FFQ (FFQ1) was conducted during postpartum at 60–65 days and the second FFQ (FFQ2) during subsequent follow-up at 5 weeks. The 3DR was completed with portion sizes assessed using photographs taken by the respondent before and after eating (instant photography) 1 week after FFQ1.

**Results:**

For reproducibility, the Spearman’s correlation coefficients for food ranged from 0.34 to 0.68, and for nutrients from 0.25 to 0.61. Meanwhile, the intra-class correlation coefficients for food ranged from 0.48 to 0.87, and for nutrients from 0.27 to 0.70. For relative validity, the Spearman’s correlation coefficients for food ranged from 0.32 to 0.56, and for nutrients from 0.23 to 0.72. The energy-adjusted coefficients for food ranged from 0.26 to 0.55, and for nutrients from 0.22 to 0.47. Moreover, the de-attenuation coefficients for food ranged from 0.34 to 0.67, and for nutrients from 0.28 to 0.77. The Bland-Altman plots also showed reasonably acceptable agreement between the two methods.

**Conclusions:**

This FFQ is a reasonably reproducible and a relative valid tool for assessing dietary intake of the Chinese lactating mothers.

## Background

Lactation is a special period for women to breastfeed their babies after childbirth. The lactating mothers need to gradually compensate for not only the energy consumed during pregnancy and childbirth, but also for the energy for promoting the recovery of their organs and system functions; additionally, they also need to secrete high-quality breast milk for the growth and development of the infant [1, 2]. Hence, the energy and nutrient requirements of the lactating mothers are higher than that of non-lactating mothers [3]. A balanced diet can ensure adequate supply of energy and nutrients; otherwise, it will have a significant impact on maternal health and infant growth and development. On the one hand, long-term unbalanced dietary intake of the lactating mothers may lead to malnutrition, especially the lack of micronutrients, such as vitamin A, iodine and iron [4]. On the other hand, excessive dietary intake can lead to postpartum weight retention, an important cause of obesity in the lactating mothers [5]. Furthermore, maternal obesity is closely related to diabetes, hypertension, depression, and other diseases [6–8]. For infants, breast milk is the most ideal food that provides sufficient energy and nutrients to ensure their growth and development. Studies have shown that maternal diet is an important factor affecting the secretion and composition of breast milk, especially water-soluble vitamins and some minerals [9, 10]. Therefore, maternal diet can affect infant development [11]. Furthermore, these effects will continue into childhood and adulthood [12, 13]. Therefore, mothers should be informed of what constitutes a healthy diet and the potential risks of nutrient deficiencies in achieving an optimal nutritional status during lactation.

Due to the influences of regional economy, traditions, and lack of knowledge, dietary nutrition problems of the lactating mothers in China are not consistent in different regions [14, 15]. Dietary assessment and evaluation are crucial for understanding how to achieve a balanced dietary intake. The 24 h-dietary recall has been used in many studies to collect dietary information of the lactating mothers [3, 16, 17]. However, these recalls generally involve a limited number of days and gather data only on short-term dietary intake [18]. The food frequency questionnaire (FFQ) is usually used to evaluate long-term dietary intake [19–21]. Studies have shown that the FFQ is the most feasible and cost-effective tool when compared to other dietary assessment methods [18]. It is relatively easy to use, inexpensive, and can help obtain data regarding the usual dietary intake and pattern of participants [21]. Therefore, it has been widely used in large-scale nutritional epidemiology studies in different populations [22]. Because dietary habits greatly vary in populations with different regional, ethnic, dietary, or cultural backgrounds, the FFQ should be tailored, reproducible, and valid for use in a specific population [22, 23].

In addition to the FFQ for general adults, FFQ for different female populations have been established in China, mainly involving girls, female adolescents, pregnant women, and postmenopausal women [24–27]. However, the special lactation period entails that the lactating mothers eat more than general adult women, wherein the number of meals a day may vary from 5 to 8 [28]. They are more likely to eat home-made food, and are discouraged from consuming beverages and snacks. In addition, on the one hand, traditional customs impose dietary restrictions on the new mother during the lactation period, by forbidding foods, such as pepper, onion, garlic, leek, hawthorn, orange, coffee, crab, shrimp, some sea fish, etc. [14, 15]. On the other hand, as compared to general adult women, the lactating mothers are encouraged to increase their intake of millet, fermented glutinous rice, jujube, egg, crucian carp, chicken, pig’s hoof, and other food items [14]. Therefore, the FFQ that can be used by either general adults or women in the special period mentioned above is not applicable to the lactating mothers in China. Therefore, we developed a semi-quantitative FFQ suitable for the Chinese lactating mothers. Moreover, we assessed the reproducibility and relative validity of the questionnaire to evaluate the dietary intake of the lactating mothers in Nanjing, China.

## Methods

### Participants

The lactating mothers at 30–50 days of postpartum were randomly recruited at their first postpartum examination in the Maternal and Child Health and Family Planning Service Centre of Jiangning District in Nanjing city. The inclusion criteria were healthy lactating mothers without infectious diseases, chronic and malignant diseases, and nutritional diseases. A subsample of 129 participants were invited to join the study from November 2018 to June 2019, in which 17 participants were excluded owing to an incomplete dietary record or FFQ. Finally, 112 lactating mothers were enrolled in the study. According to a study conducted by Willett [22], the sample size of our study can be used to assess the reproducibility and relative validity of the FFQ. This study was approved by the Ethics Committee of Nanjing Medical University (NJMU-2018-699). All the lactating mothers provided informed consent before recruitment.

### Study design

**Fig. 1:**
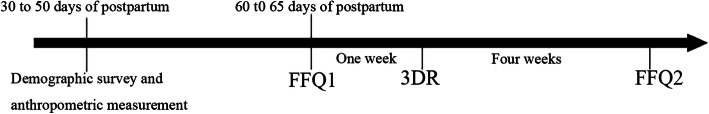
The design of the reproducibility and relative validity study among 112 lactating mothers in China

The design of the study is shown in Fig. 1. The lactating mothers were asked to complete two FFQs and one 3-d dietary record (3DR). During this period, three face-to-face interviews were conducted. In the first interview, demographic questionnaires and anthropometrics were completed and measured, respectively. The first FFQ (FFQ1) was conducted in person by a trained interviewer at 60–65 days postpartum and the second FFQ (FFQ2) at 5 weeks later during follow-ups. The 3DR was completed 1 week after the FFQ1, including two weekdays and one weekend. The time period covered by the FFQ1 and the FFQ2 was the previous month.

### Food frequency questionnaire (FFQ)

The semi-quantitative FFQ used in this study consisted of three parts, including the food list, frequency of consuming a certain food, and amount of food consumed each time. There were 156 items on the food list and were divided into 13 categories (Table 1): cereals and cereal products; potatoes; coarse cereals; vegetables; fruits; meat; poultry; aquatic products; eggs; beans and legume products; milk and milk products; nuts; and spices. Items on the list were based on the Chinese balanced dietary pagoda [29], the Chinese dietary guidelines [30], and the dietary habits of the Chinese lactating mothers, which were assessed by a research nutritionist. The frequency options provided on the FFQs were (1) none/no consumption, (2) number of times per day, (3) number of times per week, and (4) number of times per month. In order to improve the accuracy of food weight estimation for the lactating mothers, we used a photographic atlas of food portion sizes developed by our research team to facilitate the assessment of portion sizes [31]. In addition to 156 food items, the FFQ contained an open section for the lactating mothers to fill out food items that they had consumed, but were not included in the FFQ.

**Table 1:** Description of the food items included in the food categories

Food categories (Number of food items)	Food name^a^
Cereals and cereals products (9)	Rice, Rice porridge, Millet, Black rice, Wheat noodle, Wheat bun / Wheat pancake, Clay over rolls, Wheat dough stick (deep fried), Fermented glutinous rice
Potatoes (3)	Potato, Sweet potato and its products, Potato starch noodle
Coarse cereals (4)	Corn, Corn flour (yellow, dried), Buckwheat, Job’s tears
Vegetables (46)	Radish (white / red / green), Carrot (red / yellow), Cowpea / Snow pea / Kidney bean, Pale green soybean, Soybean sprouts, Sprout (Mung bean), Eggplant, Tomato, Okra, Chinese wax gourd, Calabash, Cucumber, Balsam pear, Pumpkin, Scallion, Chinese chive, Chives flowering stalk, Bok choi, Chinese cabbage, Cabbage, Cauliflower, Spinach, Celery, Endive lettuce, Coriander leaf, Amaranth, Chrysanthemum crown daisy, Shepherd’s purse, Lettuce stem, Water spinach, Seedling, Bamboo shoot, Daylily flower, Asparagus stem, Lotus root, Water bamboo, Yam, Taro, Straw mushroom / Button mushroom, Gold needle mushroom, Oyster mushroom, Wood ear fungus, Shitake mushroom, Silver ear fungus, Kelp, Laver
Fruits (28)	Apple, Pear, Peach, Plum, Apricot, Date / Date (dried), Cherry, Grape / Raisin, Persimmon, Mulberry, Chinese kiwi fruit, Strawberry, Kumquat (oval), Pomelo, Pineapple, Jackfruit flesh, Longan / Longan (dried), Jujube, Mango, Papaya, Banana, Bayberry, Coconut, Loquat, Casaba / Hami cantaloupe, Watermelon, Durian, Dragon fruit
Meat (13)	Pork, Pork (heart / liver / kidney / large intestine), Pork (ear), Pork (hoof), Pork (blood), Luncheon meat / Pork ham sausage / Pork (ham) / Pork floss / Pork (bacon), Beef, Beef (liver/tripe), Beef (tendon), Beef (tongue), Beef (dried), Lamb, Lamb (tripe / liver / large intestine)
Poultry (12)	Chicken, Chicken (wing) / Duck (wing), Chicken (leg), Chicken (feet) / Duck (feet), Chicken (liver / heart / gizzard), Chicken (blood) / duck (blood), Duck, Duck (intestine / liver / gizzard), Duck (tongue), Goose, Goose (liver / gizzard), Pigeon / Quail
Aquatic products (15)	Crucian carp, Grass carp / Black carp, Rice eel, Snakehead, Sliver carp, Goldfish carp / Common carp / Bream, Chinese perch, Yellow croaker (large / small), Abalone, Razor clam, Oyster, Scallop (fresh) / Clam, Sea cucumber (fresh), Squid (fresh), Octopus
Eggs (2)	Hen's egg, Goose's egg / Duck's egg / Quail's egg
Beans and legume Products (10)	Soybean, Tofu, Soybean milk, Soybean curd sheet (semisoft / thin strip / dried / rolled), Soybean curd slab (semisoft), Soybean milk powder, Mung bean and its product, Rea bean, Adzuki bean porridge, Other beans,
Milk and milk products (5)	Milk, Whole milk powder, Full fat, Cheese, Cream / Butter / Canned
Nuts (7)	Walnut (dried) / Wild walnut (dried), Chestnut (roasted), Pine-nut (roasted) / Almond kernel (roasted) / Cashew (roasted), Peanut, Sunflower seed (roasted) / Pumpkin seed (roasted) / Watermelon seed (roasted), Lotus seed (dried), Sesame (white and black)
Spices (2)	Salt, Cooking oil

^a^The food name comes from China food composition tables (6th edition)

### 3-d dietary record

Instant photography developed by our team [32] was used to record the 3-d normal diet of the lactating mothers. Our researchers explained to mothers how to record their diet using their phone or camera, including oral and written descriptions of the method and the video of the specific operation, in order to promote their cooperation and ensure the quality of the work. Before each meal, the food (rice, vegetables, soup, etc.) of the lactating mothers was to be placed on a separate flat table to ensure that they were not mixed with the food of other family members. Then, the food was to be placed in the red area of the two-dimensional background (scale with 1 cm × 1 cm). A note was suggested to place the background information next to the food, indicating the name of the food, the ingredients of the food mix, and the added cooking oil and salt. Next, the food was to be photographed directly from above, at 45° both front and back. The participants were instructed that the whole red area of the background should also appear in all photographs. After the meal, the same method was used to photograph the unfinished food. Finally, the photos were sent to the assessors to estimate the amount of food intake. Dietary records of the mothers were checked when received. For missing or unclear photos, mothers were asked to retake the food pictures to ensure the reliability and the relative validity of the results.

### Food and nutrient assessment

Primary data obtained from two FFQs and one 3DR were double entered into the EpiData software by two trained assessors to verify the accuracy. For food items in the FFQs, the total daily food consumption was calculated by multiplying the frequency of daily food consumption and the amount of food consumed each time. For food items in the 3DR, the food photos were compared with the food atlas of instant photography to estimate the intake of each food [33]. The daily food consumption and nutrient intakes in the two FFQs and one 3DR were calculated according to the China Food Composition Tables (6th edition) [34]. Data of all food groups and nutrient intakes were imported to Microsoft Excel for statistical analysis.

### Statistical analysis

All data were calculated and analysed using the Statistical Package for the Social Sciences version 26.0. A *p* value of < 0.05 was considered significant. Categorical variable data, such as body mass index (BMI) grade, educational status, mode of delivery, and parity were represented by frequency (n) and percentage (%). Normally - continuous variable data, such as maternal age, height, weight, and BMI, were represented by mean and standard deviation (SD). The data distribution of daily food consumption and nutrient intakes from two FFQs and one 3DR were non-normal, and thus, were described by median, 25th-, and 75th percentiles, respectively. Differences in food consumption and nutrient intake between two FFQs, and between the FFQ (average of two FFQs) and the 3DR (average of 3 days), were compared using the Wilcoxon signed rank test.

The Spearman rank correlation coefficients and intra-class correlation coefficients were calculated to test the reproducibility between the FFQ1 and FFQ2. The Spearman rank correlation coefficients were estimated to test the relative validity between the FFQ (average of two FFQs) and the 3DR (average of 3 days). Since most of the food and nutrient intake was related to the total energy intake, Willett’s residual method [35] was used to calculate the energy-adjusted intake of each food and nutrient. Moreover, the energy-adjusted correlation coefficient was calculated to assess the relationship between the FFQ (average of two FFQs) and the 3DR (average of 3 days). Due to the influence of inter-individual and intra-individual variation, the correlation coefficient after the elimination of individual variation was calculated according to the formula: R_de_ = r(1 + λ/n_x_)^0.5^, where λ is the ratio of intra-individual variation and inter-individual variation, n_x_ is the number of repeated days [36], and n_x_ is 3 in the study.

Quartile agreement was used to test the reproducibility and relative validity of the FFQ. Specifically, quartile agreements between the two FFQs, and between the FFQ (average of two FFQs) and the 3DR (average of 3 days), were calculated based on the percentage of the lactating mothers classified in the same, adjacent, and extreme quartiles. In addition, on the premise that the distribution of different between FFQ and 3 DR was normal, the Bland–Altman plots were used to examine the agreement between the FFQ (average of two FFQs) and the 3DR (average of 3 days) for food categories and nutrients [37]. Furthermore, calculating for the difference between the two methods and for the 95% reference range of the difference were essential for observing the dispersion trend of the difference and for investigating the 95% limits of agreement (LOAs), respectively.

## Results

### Characteristics of the lactating mothers

The characteristics of the lactating mothers are shown in Table 2. The average age was 29.4 years and the average BMI was 22.9 kg/m^2^. According to the BMI standard of Chinese adults [38], 4.5% were underweight (BMI < 18.5 kg/m^2^), 57.1% were normal (BMI = 18.5–23.9 kg/m^2^), 33.9% were overweight (BMI = 24.0 ~ 27.9 kg/m^2^), and 4.5% were obese (BMI > 28.0 kg/m^2^). Most lactating mothers (58.9%) had university degrees or above, and most of them (78.6%) were professionals. Moreover, more than half (62.5%) gave birth naturally, and 65.2% had given birth only once before.

**Table 2:** Demographic and anthropometric characteristics of 112 lactating mothers

Characteristics	Results
Age, y ($\overline{X}\pm \mathrm{SD}$)	29.4 ± 3.9
Height, cm ($\overline{X}\pm \mathrm{SD}$)	161.4 ± 4.9
Weight, kg ($\overline{X}\pm \mathrm{SD}$)	60.8 ± 8.0
BMI, kg/m^2^ ($\overline{X}\pm \mathrm{SD}$)	22.9 ± 4.1
Underweight (n, %)	5 (4.5)
Normal weight (n, %)	64 (57.1)
Overweight (n, %)	38 (33.9)
Obesity (n, %)	5 (4.5)
Education (n, %)	
Junior high school	5 (4.5)
Senior high school	16 (14.3)
Vocational-technical school	25 (22.3)
University degree or above	66 (58.9)
Employment status (n, %)	
Employed	24 (21.4)
Unemployed	88 (78.6)
Delivery way (n, %)	
Spontaneous labor	70 (62.5)
Others	42 (37.5)
Parity (n, %)	
Once	73 (65.2)
Twice or more	39 (34.8)

### Reproducibility

The median daily intake of food groups from the FFQ1 and FFQ2 showed that consumption of cereals and cereal products, potatoes, beans and legume products, milk, and nuts was higher, whereas that of vegetables, fruits, meat, aquatic products, and eggs was lower, when estimated by FFQ2 than by FFQ1. Further statistical analysis showed that there were no significant differences in the consumption of the food categories, except meat, between the two FFQs. The spearman rank correlation coefficient ranged from 0.34 for nuts, to 0.68 for poultry, with an average of 0.46. Furthermore, all correlations were statistically significant (*p* < 0.01). Similarly, the intra-class correlation coefficients ranged from 0.48 for cereals and cereal products, to 0.87 for poultry, with an average of 0.61. Similarly, all correlations were also statistically significant (*p* < 0.01). The percentage of agreement (the same or adjacent quartile) between the food intakes in both FFQs was 62.6% on average and ranged from 50.4% for eggs to 70.8% for potatoes (Table 3).

**Table 3:** Reproducibility study: Median daily food intakes, correlation coefficients and the agreement between FFQ1and FFQ2 in 112 lactating mothers

Food groups	Intake value Median (P25, P75)		*p –*value^c^	Correlation coefficient		Agreement		
						Percentage classified in		
	FFQ1^a^	FFQ2^b^		r_s_^d^	r_i_^e^	Same quartile	Same or adjacent quartile	Extreme quartile
Cereals and cereal products (g/day)	145 (108,185)	153 (126,192)	0.10	0.41^**^	0.48^**^	22.1	55.7	30.1
Potatoes (g/day)	15 (1.5,35.5)	20 (1.5,40)	0.56	0.38^**^	0.57^**^	31.0	70.8	10.6
Coarse cereals (g/day)	15 (1,32.5)	15 (3,26)	0.47	0.38^**^	0.59^**^	27.4	64.6	10.6
Vegetables (g/day)	310 (201,416)	295 (207,434)	0.92	0.47^**^	0.64^**^	30.1	60.2	13.3
Fruits (g/day)	310 (205,430)	305 (227,391)	0.79	0.45^**^	0.64^**^	26.5	64.6	15.0
Meat (g/day)	100 (63,140)	80 (57,108)	0.008	0.50^**^	0.61^**^	21.2	63.7	9.7
Poultry (g/day)	60 (40,80)	60 (40,79)	0.83	0.68^**^	0.87^**^	20.4	67.3	10.6
Aquatic products (g/day)	45 (30,93)	40 (27,73)	0.25	0.42^**^	0.50^**^	27.4	66.3	13.3
Eggs (g/day)	60 (49,72)	58 (50,77)	0.59	0.61^**^	0.69^**^	11.5	50.4	17.7
Beans and legume products (g/day)	9 (3,17)	10 (5,20)	0.26	0.43^**^	0.65^**^	23.0	60.2	9.7
Milk and milk products (g/day)	134 (0,234)	167 (42,201)	0.94	0.39^**^	0.55^**^	23.9	65.5	15.0
Nuts (g/day)	8 (0,20)	10 (0,20)	0.39	0.34^**^	0.57^**^	27.4	61.9	15.0

^a^ FFQ1: the first food frequency questionnaire which was conducted at 60–65 days postpartum

^b^ FFQ2: the second food frequency questionnaire which was conducted at 5 weeks later during follow-ups

^c^ *p*-value: Wilcoxon signed rank test

^d^ r_s_: Spearman rank correlation coefficients

^e^ r_i_: Intra-class correlation coefficients

^*^p < 0.05, ^**^p < 0.01

The median daily intake of energy and nutrients from FFQ1 and FFQ2 showed that consumption of protein, fat, total fatty acid, saturated fatty acid (SFA), monounsaturated fatty acid (MUFA), polyunsaturated fatty acid (PUFA), n-6 polyunsaturated fatty acid (n-6 PUFA), dietary fibre, vitamin A, niacin, and iron was lower when estimated by FFQ2 than by FFQ1; other nutrients estimated by FFQ2 were slightly higher than or equal to those estimated by FFQ1. Further statistical analysis showed that there were no significant differences in energy and nutrient consumption between the two FFQs, except for vitamin A. The Spearman rank correlation coefficients ranged from 0.25 for vitamin A, to 0.61 for protein, with an average of 0.43, and all correlations were statistically significant (*p* < 0.01). Similarly, the intra-class correlation coefficients ranged from 0.27 for vitamin A, to 0.70 for energy, with an average of 0.44, and all correlations were statistically significant (*p* < 0.05). The percentage of agreement (the same or adjacent quartile) of energy and nutrient intake in both FFQs was 80.4% on average and ranged from 73.5% for vitamin A, to 88.5% for phosphorus (Table 4).

**Table 4:** Reproducibility study: Median daily energy and nutrient intakes, correlation coefficients and agreement between FFQ1and FFQ2 in 112 lactating mothers

Energy and nutrients	Intake value Median (P25, P75)		*p* –value^c^	Correlation coefficient		Agreement		
						Percentage classified in		
	FFQ1^a^	FFQ2^b^		r_s_^d^	r_i_^e^	Same quartile	Same or adjacent quartile	Extreme quartile
Energy (kcal/day)	2006.2 (1632.9, 2229.3)	2009.4 (1635.7, 2231.4)	0.91	0.59^**^	0.70^**^	65.5	84.1	6.2
Protein (g/day)	85.4 (69.1, 103.9)	84.5 (68.1, 99.5)	0.68	0.61^**^	0.55^**^	46.9	82.3	5.3
Fat (g/day)	88.3 (73.1, 98.7)	83.0 (70.9, 95.5)	0.19	0.44^**^	0.34^**^	43.4	83.2	7.1
Total Fatty acid (g/d)	68.6 (47.7, 86.5)	61.5 (43.9, 82.7)	0.17	0.43^**^	0.45^**^	43.7	82.2	7.2
SFA (g/d) ^f^	14.5 (8.0, 22.7)	11.6 (7.2, 17.6)	0.06	0.41^**^	0.33^*^	48.2	80.4	8.1
MUFA (g/d) ^g^	25.3 (15.0, 36.2)	21.9 (13.4, 31.9)	0.11	0.39^**^	0.38^**^	44.6	81.3	8.1
PUFA (g/d) ^h^	11.9 (7.0, 17.7)	10.4 (6.6, 17.0)	0.73	0.43^**^	0.41^**^	46.4	83.2	6.3
n-3 PUFA (g/d) ^i^	0.06 (0.04, 0.10)	0.06 (0.04, 0.09)	0.14	0.35^**^	0.28^*^	41.9	76.9	8.1
n-6 PUFA (g/d) ^j^	0.80 (0.50, 1.14)	0.76 (0.50, 1.01)	0.17	0.29^**^	0.33^*^	51.8	78.8	10.8
Carbohydrate (g/d)	212.5 (171.6, 250.8)	216.4 (170.4, 260.4)	0.41	0.50^**^	0.55^**^	45.1	83.2	5.3
Dietary fiber (g/d)	15.0 (10.7, 20.5)	14.8 (10.9, 19.8)	0.85	0.33^**^	0.36^**^	43.6	77.9	8.8
Vitamin A (μgRAE/day) ^k^	857.2 (597.3, 1330.3)	721.5 (559.2, 976.9)	0.04	0.25^**^	0.27^*^	41.6	73.5	10.6
Thiamin (mg/day)	0.9 (0.7, 1.1)	0.9 (0.7, 1.1)	0.69	0.53^**^	0.47^**^	51.3	84.9	4.4
Riboflavin (mg/day)	1.3 (1.1, 1.7)	1.3 (1.1, 1.5)	0.17	0.42^**^	0.29^**^	46.0	74.3	8.0
Niacin (mg/day)	20.3 (15.9, 26.9)	20.0 (15.3, 24.5)	0.21	0.57^**^	0.50^**^	50.4	82.3	2.7
Vitamin C (mg/day)	135.4 (78.6, 194.1)	139.6 (96.6, 194.0)	0.67	0.31^**^	0.43^**^	38.9	75.2	8.0
Vitamin E (mg/day)	34.0 (30.3, 40.6)	35.9 (30.9, 41.9)	0.38	0.30^**^	0.32^**^	46.0	77.9	8.0
Calcium (mg/day)	625.4 (501.3, 789.9)	706.0 (542.2, 845.7)	0.13	0.39^**^	0.53^**^	38.9	77.0	6.2
Phosphorus (mg/day)	1261.1 (1041.8, 1495.3)	1304.6 (1056.1, 1500.3)	0.68	0.59^**^	0.52^**^	45.1	88.5	2.7
Potassium (mg/day)	2555.9 (2105.9, 3258.7)	2568.9 (2126.1, 3298.8)	0.93	0.47^**^	0.59^**^	52.2	80.5	3.5
Magnesium (mg/day)	356.5 (286.1, 431.9)	362.5 (289.2, 446.3)	0.59	0.44^**^	0.50^**^	43.4	78.8	8.0
Iron (mg/day)	25.1 (20.0, 31.8)	24.9 (20.5, 31.2)	0.77	0.45^**^	0.32^**^	51.3	80.5	8.0
Zinc (mg/day)	12.9 (10.6, 15.8)	13.1 (10.5, 15.7)	0.92	0.58^**^	0.66^**^	52.2	87.6	4.4
Selenium (μg/day)	58.5 (50.7, 73.2)	58.7 (47.7, 69.9)	0.59	0.26^**^	0.29^*^	38.9	75.2	9.7
Copper (mg/day)	2.4 (1.8, 3.0)	2.5 (2.0, 3.1)	0.71	0.48^**^	0.44^**^	49.6	78.8	4.4
Manganese (mg/day)	4.9 (4.0, 6.1)	5.2 (4.0, 6.7)	0.23	0.44^**^	0.48^**^	46.9	81.4	5.3

^a^ FFQ1: the first food frequency questionnaire which was conducted at 60–65 days postpartum

^b^ FFQ2: the second food frequency questionnaire which was conducted at 5 weeks later during follow-ups

^c^ *p* -value: Wilcoxon signed rank test

^d^ r_s_: Spearman rank correlation coefficients

^e^ r_i_: Intra-class correlation coefficients

^f^ SFA: saturated fatty acid

^g^ MUFA: monounsaturated fatty acid

^h^ PUFA: polyunsaturated fatty acid

^I^ n-3 PUFA: n-3 polyunsaturated fatty acid

^j^ n-6 PUFA: n-6 polyunsaturated fatty acid

^k^ RAE: retinol activity equivalent

^*^p < 0.05, ^**^p < 0.01

### Relative validity

As shown in Table 5, the median daily intakes of food groups from the FFQs, except for cereals and cereal products, meat, poultry, and eggs, were higher than those from the 3DR. Further statistical analysis showed that there were significant differences in the intake of cereals and cereal products, vegetables, fruits, meat, and nuts, as assessed by the FFQs and 3DR. The unadjusted spearmen rank correlation coefficients ranged from 0.32 for nuts, to 0.56 for cereals and cereal products, with an average of 0.45, and all correlations were statistically significant (*p* < 0.05). Majority of the correlation coefficients for food groups decreased or remained unchanged after energy adjustment; however, those for potatoes and poultry increased slightly. The energy-adjusted correlation coefficient ranged from 0.26 for nuts, to 0.55 for cereals and cereal products, with an average of 0.43, and all correlations were statistically significant (*p* < 0.01). The average de-attenuated correlation coefficients were 0.46, ranging from 0.34 for milk and milk products, to 0.67 for cereals and cereals products, and all correlations were also statistically significant (*p* < 0.05). The percentage of agreement (the same or adjacent quartile) of food intake obtained from both the FFQs and 3DR was 69.0% on average and ranged from 61.2% for coarse cereals, to 83.2% for eggs, beans and legume products.

**Table 5:** Relative validity study: Median daily food intakes, correlation coefficients and agreement between FFQ and 3DR in 112 lactating mothers

Food groups	Intake value Median (P25, P75)		*p* -value ^c^	Correlation coefficient			Agreement		
							Percentage classified in		
	FFQ ^a^	3DR ^b^		r ^d^	r _e-adj_^e^	r _de-att_^f^	Same quartile	Same or adjacent quartile	Extreme quartile
Cereals and cereal products (g/day)	153 (120, 191)	192 (140, 217)	< 0.01	0.56^**^	0.55^**^	0.67^**^	25.7	62.3	13.3
Potatoes (g/day)	17 (9, 32.5)	15 (1.7, 41)	0.328	0.51^**^	0.52^**^	0.48^**^	19.5	62.9	6.2
Coarse cereals (g/day)	17.5 (6, 28)	13 (2, 39)	0.134	0.40^**^	0.37^**^	0.38^**^	23.9	61.2	8.0
Vegetables (g/day)	303 (215, 421)	294 (207, 410)	< 0.01	0.45^**^	0.45^**^	0.44^**^	31.9	71.7	8.8
Fruits (g/day)	306 (228, 411)	228 (118, 356)	< 0.01	0.38^**^	0.37^**^	0.42^**^	25.7	62.0	15.9
Meat (g/day)	95 (68, 130)	116 (73, 163)	< 0.01	0.41^**^	0.39^**^	0.50^**^	27.5	62.0	8.8
Poultry (g/day)	53 (40, 78)	60 (40, 80)	0.58	0.52^**^	0.53^**^	0.51^**^	26.5	63.7	11.5
Aquatic products (g/day)	52 (34, 79)	43 (25, 69)	0.47	0.41^**^	0.32^**^	0.47^**^	41.6	79.7	9.7
Eggs (g/day)	60 (50, 73)	68 (50, 90)	0.068	0.50^*^	0.42^**^	0.49^**^	43.4	83.2	3.5
Beans and legume products (g/day)	10 (5, 19)	6 (0, 16)	0.214	0.54^*^	0.54^**^	0.47^**^	42.5	83.2	4.4
Milk and milk products (g/day)	130 (69, 201)	83 (0, 194)	0.007	0.43^**^	0.42^**^	0.34^*^	45.1	74.3	8.0
Nuts (g/day)	10 (5, 20)	0 (0, 7.5)	< 0.01	0.32^**^	0.26^**^	0.36^**^	26.5	62.0	12.4

^a^ FFQ: average of the FFQ1 and the FFQ2

^b^ 3DR: 3-day dietary records which was completed one week after the FFQ1, including two weekdays and one weekend

^c^ *p* -value: Wilcoxon signed rank test

^d^ r: Spearman rank correlation coefficients

^e^ r _e-adj_: Energy-adjusted correlation coefficient

^f^ r _de-att_: de-attenuated correlation coefficients

^*^p < 0.05, ^**^p < 0.01

Table 6 shows the relative validity of energy and nutrients between the FFQs and the 3DR. The median daily intakes of energy and nutrients from both FFQs, except for carbohydrate and selenium, were higher than those from the 3DR. Further statistical analysis showed that there were significant differences in the intakes of fat, total fatty acid, PUFA, dietary fibre, thiamine, vitamin C, vitamin E, calcium, potassium, magnesium, and selenium, as assessed by the FFQ and 3DR. The average of unadjusted Spearmen rank correlation was 0.49 (from 0.23 for vitamin C to 0.72 for energy), and all correlations were statistically significant (*p* < 0.05). After energy adjustment, correlation coefficients of all nutrients decreased. The energy-adjusted correlation coefficient ranged from 0.22 (vitamin C) to 0.47 (phosphorus) with an average of 0.32, and all correlations were statistically significant (*p* < 0.05). Among them, the biggest change was that of proteins, where its correlation coefficient decreased from 0.67 to 0.31. The average de-attenuated correlation coefficients were 0.53 (from 0.28 for vitamin C to 0.77 for energy), and all correlations were statistically significant (*p* < 0.01). The percentage of agreement (the same or adjacent quartile) of energy and nutrients intake obtained from the FFQs and the 3DR was 81.1% on average and ranged from 68.1% for vitamin C to 95.5% for proteins.

**Table 6:** Relative validity study: Median daily energy and nutrient intakes, correlation coefficients and agreement between FFQ and 3DR in 112 lactating mothers

Energy and nutrients	Intake value Median (P25, P75)		*p*- value ^c^	Correlation coefficient			Agreement		
							Percentage classified in		
	FFQ ^a^	3DR ^b^		r ^d^	r _e-adj_^e^	r _de-att_^f^	Same quartile	Same or adjacent quartile	Extreme quartile
Energy (kcal/day)	2017.7 (1712.9, 2222.4)	1992.4 (1636.3, 2232.8)	0.87	0.72^**^		0.77^**^	62.8	91.1	4.4
Protein (g/day)	85.7 (70.1, 101.6)	83.4 (71.9, 100.6)	0.72	0.67^**^	0.31^**^	0.71^**^	72.4	95.5	0
Fat (g/day)	86.1 (73.3, 94.8)	77.7 (62.5, 93.2)	0.028	0.51^**^	0.33^**^	0.57^**^	42.5	85.1	4.4
Total Fatty acid (g/d)	66.3 (40.7, 80.3)	51.6 (33.9, 74.5)	0.02	0.48^**^	0.28^**^	0.54^**^	40.2	84.9	4.5
SFA (g/d) ^g^	14.8 (8.8, 19.3)	9.5 (5.2, 18.3)	0.25	0.46^**^	0.28^**^	0.53^**^	37.5	80.4	4.5
MUFA (g/d) ^h^	25.6 (15.3, 2.3)	17.6 (9.1, 29.5)	0.055	0.46^**^	0.28^**^	0.53^**^	40.2	82.1	5.4
PUFA (g/d) ^i^	11.8 (7.6, 17.3)	7.6 (4.3, 11.9)	0.001	0.50^**^	0.24^*^	0.51^**^	36.5	81.2	5.4
n-3 PUFA (g/d) ^j^	0.07 (0.04, 0.09)	0.05 (0.03, 0.10)	0.619	0.44^**^	0.27^**^	0.43^**^	36.6	79.4	4.5
n-6 PUFA (g/d) ^k^	0.80 (0.59, 1.08)	0.71 (0.49, 1.42)	0.187	0.49^**^	0.28^**^	0.54^**^	29.5	83.8	3.6
Carbohydrate (g/d)	217.3 (175.7, 249.1)	222.6 (177.3, 272.0)	0.272	0.71^**^	0.45^**^	0.76^**^	50.4	90.2	2.7
Dietary fiber (g/d)	15.6 (11.8, 20.3)	12.7 (9.7, 16.2)	0.002	0.44^**^	0.34^**^	0.57^**^	38.9	78.7	8.0
Vitamin A (μgRAE/day) ^l^	890.1 (636.1, 1151.6)	842.9 (569.11200.0)	0.90	0.26^**^	0.24^**^	0.32^**^	30.1	73.4	8.0
Thiamin (mg/day)	0.9 (0.8, 1.1)	0.9 (0.8, 1.2)	0.036	0.62^**^	0.39^**^	0.68^**^	54.0	81.4	1.8
Riboflavin (mg/day)	1.4 (1.2, 1.6)	1.3 (1.1, 1.7)	0.69	0.44^**^	0.30^**^	0.53^**^	41.6	80.5	7.1
Niacin (mg/day)	21.1 (16.4, 25.6)	20.1 (14.9, 25.8)	0.65	0.58^**^	0.41^**^	0.66^**^	44.2	84.9	3.5
Vitamin C (mg/day)	137.9 (105.5, 186.2)	109.3 (78.6, 151.3)	< 0.01	0.23^*^	0.22^*^	0.28^**^	38.9	68.1	7.1
Vitamin E (mg/day)	36.5 (31.9, 41.4)	26.7 (23.4, 31.9)	0.005	0.43^**^	0.29^*^	0.37^**^	34.5	76.1	3.5
Calcium (mg/day)	667.4 (546.9, 824.1)	605.7 (432.8, 713.8)	0.013	0.31^**^	0.24^*^	0.41^**^	37.2	70.8	6.2
Phosphorus (mg/day)	1292.5 (1090.6, 1508.8)	1213.3 (1016.5, 1401.4)	0.051	0.63^**^	0.47^**^	0.69^**^	50.4	84.9	2.7
Potassium (mg/day)	2611.8 (2233.9, 3243.3)	2332.7 (1861.1, 2617.2)	0.003	0.44^**^	0.36^**^	0.43^**^	38.1	76.2	4.4
Magnesium (mg/day)	364.1 (306.1, 435.9)	329.8 (277.0, 382.3)	0.027	0.49^**^	0.33^**^	0.41^**^	38.1	84.1	1.8
Iron (mg/day)	26.1 (21.3, 30.5)	24.7 (21.3, 29.7)	0.43	0.39^**^	0.24^*^	0.44^**^	42.5	78.9	5.3
Zinc (mg/day)	13.4 (10.9, 15.7)	13.0 (10.4, 15.6)	0.51	0.64^**^	0.42^**^	0.68^**^	55.8	87.6	2.7
Selenium (μg/day)	61.4 (53.1, 69.7)	62.4 (48.7, 82.7)	0.021	0.41^**^	0.30^**^	0.32^**^	34.5	76.1	3.5
Copper (mg/day)	2.6 (2.0, 3.0)	2.1 (1.7, 2.8)	0.53	0.41^**^	0.27^**^	0.51^**^	31.9	76.1	4.4
Manganese (mg/day)	5.3 (4.3, 6.4)	5.4 (4.1, 6.2)	0.91	0.57^**^	0.46^**^	0.62^**^	49.6	77.9	1.8

^a^ FFQ: average of the FFQ1 and the FFQ2

^b^ 3DR: 3-day dietary records which was completed one week after the FFQ1, including two weekdays and one weekend

^c^ *p* –value: Wilcoxon signed rank test

^d^ r: Spearman rank correlation coefficients

^e^ r _e-adj_: Energy-adjusted correlation coefficient

^f^ r _de-att_: de-attenuated correlation coefficients

^g^ SFA: saturated fatty acid

^h^ MUFA: monounsaturated fatty acid

^i^ PUFA: polyunsaturated fatty acid

^j^ n-3 PUFA: n-3 polyunsaturated fatty acid

^k^ n-6 PUFA: n-6 polyunsaturated fatty acid

^l^ RAE: retinol activity equivalent

^*^p < 0.05, ^**^p < 0.01

### Bland-Altman analysis

**Fig. 2:**
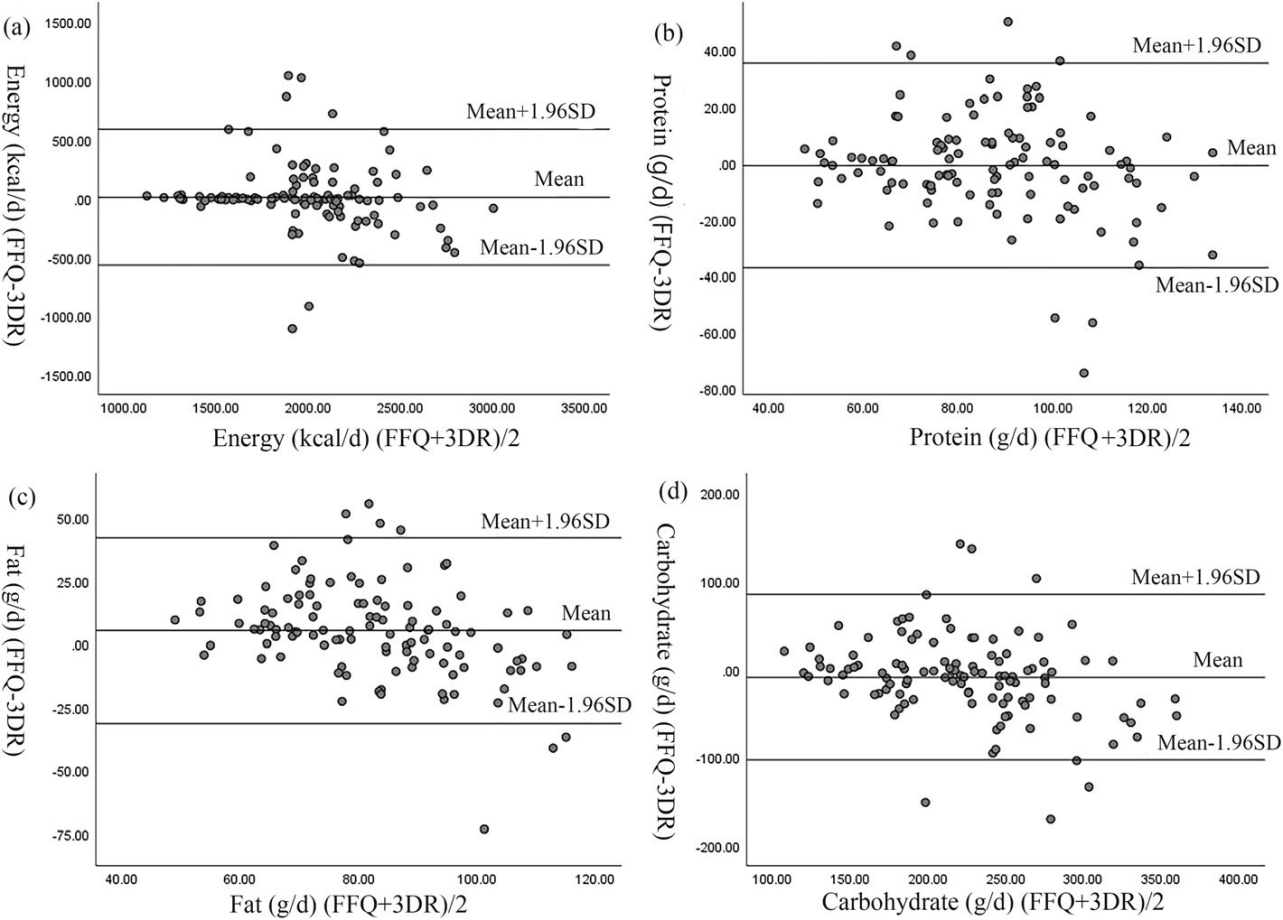
Bland-Altman plots showing the relationship between mean and differences in the daily intake of (**a**) energy, (**b**) protein, (**c**) fat and (**d**) carbohydrate between FFQ and 3DR in 112 lactating mothers

Agreements between the food groups and the energy and nutrients from both FFQs and 3DR were determined using the Bland-Altman plots (Fig. 2), which shows the relationship between the mean and the difference in the daily intakes of energy, protein, fat, and carbohydrate obtained from both FFQs and 3DR. The x-axis represents the mean total intake of energy and macronutrients from both FFQs and 3DR, whereas the y- axis represents the difference of energy and macronutrients intake between the two methods. A good agreement was defined as having no more than 10% of the points exceeding the 95% LOAs and were close to the mean line. As shown in Fig. 2, except for some points outside the 95% LOAs, most of the points were within the LOAs and most of them were close to the mean line.

## Discussion

We assessed the reproducibility and relative validity of a semi-quantitative 156-item FFQ, which was designed to evaluate the dietary intake of the lactating mothers in China. In our study, the first FFQ was performed after 60 to 65 days postpartum. The FFQ is used to survey a person’s dietary behaviour over a period of time. The interval of retest reproducibility ranged from a week to several years in different studies. Compared to the general population, the lactating mothers spend a lot of time on infant care, resulting in a lack of adequate sleep. If the interval between the two FFQs was relatively long, serious recall bias may be warranted. Therefore, the second FFQ was completed 5 weeks later during follow-ups, in which each FFQ recorded the participant’s diet for the previous month.

In the reproducibility study, the average Spearman rank correlation coefficients were 0.46 for food, and 0.43 for energy and nutrients, whereas the average intra-class correlation coefficients were 0.61 for food, and 0.44 for energy and nutrients. Similar to the reports of a previous study, most of the correlation coefficients in this study were above 0.4, thereby showing a good correlation [39]. Among them, the correlation coefficients of eggs and poultry were higher than those of the others. Moreover, this finding was also confirmed in FFQ studies from pregnant women and children aged 7–9 years [40, 41]. A possible reason for the higher correlation coefficients of eggs and poultry could be that these special populations had special physiologic needs, resulting in an increase in the demand for high-quality protein; eggs and poultry are good sources of high-quality protein. Chinese dietary culture entails increasing the intake of eggs and poultry for the lactating mothers. However, as compared to other people with special physiologic needs, the diet of the lactating women has its particularity. For example, as compared to the correlation coefficient of fruits in the FFQ from pregnant women in previous studies [42, 43], the results in our study was relatively low. A reason for such may be due to the dietary taboo wherein the lactating mothers should eat less raw or cold food, such as fruits. Moreover, as compared to the FFQ studies from some coastal countries [44, 45], the correlation coefficients of some fatty acids in our study were low. A reason for this may be that the investigated region was an inland city of China, where consumption of marine food was less. Moreover, the correlation coefficients of vitamin A, vitamin C, iron, and selenium were also low in our study. A possible reason could be that the food sources with these nutrients were relatively limited. For example, animal liver, dark-green leafy vegetables, and fruits are rich in vitamin A. If these kinds of food were not eaten at the time of investigation, the intake levels of vitamin A of the lactating mothers would be greatly different.

As for the relative validity evaluation of the FFQ, previous studies mostly used 24 h- dietary recalls as a reference method [43, 46, 47]. In this study, instant photography was used as the reference method to record the 3-d dietary intake of the lactating mothers. Our previous study [32] found that when compared with the conventional 24 h-dietary recalls, instant photography could obtain the data of both food consumption and nutrient intake closer to the data obtained by the weighing method. Therefore, results obtained using instant photographs to assess portions were likely to have a higher degree of accuracy, improving the strength of the relative validation analysis.

Through statistical and validity analysis, we found that the average unadjusted spearmen rank correlation coefficients were 0.45 for food, and 0.49 for energy and nutrients. The correlation coefficients of most food and nutrients ranged from 0.4 to 0.7, showing moderate agreement. Similar to a previous FFQ study [48], correlation coefficients of most food and nutrients in this study decreased after energy adjustment. Specifically, the average correlation coefficient of food and nutrients decreased from 0.45 to 0.43 and from 0.49 to 0.32, respectively. This may be due to the large differences in energy intake among individuals. Similarly, variability was associated with an overestimation or an underestimation of systematic errors. In this study, due to the correction for changes in the daily intakes, the de-attenuated correlation coefficients increased, which was similar to the FFQ reliability studies from other special populations in China [23, 43]. In addition to the correlation coefficients, we used quartile cross-classification agreements and the Bland-Altman plots to evaluate the relative validity of the two methods. The percentages of agreement (the same or adjacent quartile) of food and energy, and nutrient intakes obtained from the FFQs and the 3DR, were 69.0 and 81.1%, respectively. Furthermore, the average percentages of the opposite quartile between the two methods for the intakes food and energy and nutrients were 9.2 and 4.3, respectively. These results were similar to those of previous studies on the FFQs from pregnant women [25, 49]. According to Masson’s study [50], when more than 10% of the participants were placed in the extreme quartile, the results were unsatisfactory. The misclassification rates of most food and nutrients in our study were less than 10%, which indicated that this study had good quartile agreement. On the Bland–Altman plots, overestimated or underestimated data could be clearly reflected. The closer the mean difference between the two methods was to zero, the narrower was the consistency interval. This indicated better agreement between the two methods [37]. The Bland-Altman plots showed that the agreements of most foods and nutrients between the FFQs and 3DR were satisfactory.

Our study had some limitations. First, as compared to previous FFQs with fewer items, the FFQs used in this study contained 156 food items, which covered a wide variety of foods and seasonal variations in food consumption. As a result, this study was more specific and more accurate in dietary evaluation. However, the increase in food items meant that the lactating mothers needed more time to complete the FFQs, which would have increased the pressure of field investigation. Second, during the FFQ survey, we used face-to-face interviews and a food atlas to help the lactating mothers recall the types of food they ate and to improve the accuracy of food weight estimation. Therefore, whether it could be used in self-administered settings needs further verification. Third, we also took quality control measures in the process of design, implementation, and data collection to minimize bias. However, the dietary survey itself was complex and was coupled with a special group of lactating mothers who needed postnatal recovery and infant care. These conditions could have caused inevitable bias in data reporting. Finally, the FFQ assessed in this study is a localized data-collection method that can be used to assess the local diet of lactating mothers in Nanjing or other developed cities in Southeast China. Due to regional variations in local foods, the results may not be transferable to lactating mothers in other regions.

## Conclusions

To our knowledge, this was the first FFQ specially designed for the Chinese lactating mothers. The results showed that our FFQ exhibited acceptable reproducibility and reasonable relative validity in assessing most food and nutrient intakes among the lactating mothers. Based on the present study, this FFQ may likely be applied on epidemiological investigations of the relationship between dietary intakes of the lactating mothers and related health problems in Nanjing. However, as compared to other regions in China, Nanjing can only represent the economically-developed regions in eastern China. Therefore, further work is needed to validate this FFQ for use in larger-scale surveys of the dietary intakes of the lactating mothers in different regions of China.

## Data Availability

The datasets used and analysed during the current study are available from the corresponding author on reasonable request.

## Abbreviations

FFQ: Food frequency questionnaire
3DR: 3-d dietary record
SFA: Saturated fatty acid
MUFA: Monounsaturated fatty acid
n-6 PUFA: n-6 polyunsaturated fatty acid
BMI: Body mass index
LOA: Limits of agreement
